# Phenomewide Association Study of Health Outcomes Associated With the Genetic Correlates of 25 Hydroxyvitamin D Concentration and Vitamin D Binding Protein Concentration

**DOI:** 10.1017/thg.2024.19

**Published:** 2024-04-22

**Authors:** Hailey A. Kresge, Freida Blostein, Slavina Goleva, Clara Albiñana, Joana A. Revez, Naomi R. Wray, Bjarni J. Vilhjálmsson, Zhihong Zhu, John J. McGrath, Lea K. Davis

**Affiliations:** 1 Vanderbilt Genetics Institute, Vanderbilt University Medical Center, Nashville, TN, USA; 2 National Centre for Register-Based Research, Aarhus University, Aarhus V, Denmark; 3 Department of Psychiatry, University of Oxford, Oxford, UK; 4 Institute for Molecular Bioscience, University of Queensland, Brisbane, QLD, Australia; 5 Queensland Brain Institute, University of Queensland, Brisbane, QLD, Australia; 6 Bioinformatics Research Centre, Aarhus University, Aarhus C, Denmark; 7 Novo Nordisk Foundation Center for Genomic Mechanisms of Disease, Broad Institute, Cambridge, MA, USA,; 8 Queensland Centre for Mental Health Research, The Park Centre for Mental Health, Wacol, QLD, Australia,; 9 Division of Genetic Medicine, Vanderbilt University Medical Center, Nashville, TN, USA,; 10 Division of Neurology, Pharmacology and Special Education, Vanderbilt Kennedy Center, Vanderbilt University Medical Center, Nashville, TN, USA,; 11 Department of Biomedical Informatics, Vanderbilt University Medical Center, Nashville, TN, USA; 12 Department of Psychiatry and Behavioral Sciences, Vanderbilt University Medical Center, Nashville, TN, USA; 13 Department of Molecular Physiology and Biophysics, Vanderbilt University, Nashville, TN, USA

**Keywords:** Phenomewide association study, Vitamin D, Vitamin D binding protein

## Abstract

While it is known that vitamin D deficiency is associated with adverse bone outcomes, it remains unclear whether low vitamin D status may increase the risk of a wider range of health outcomes. We had the opportunity to explore the association between common genetic variants associated with both 25 hydroxyvitamin D (25OHD) and the vitamin D binding protein (DBP, encoded by the *GC* gene) with a comprehensive range of health disorders and laboratory tests in a large academic medical center. We used summary statistics for 25OHD and DBP to generate polygenic scores (PGS) for 66,482 participants with primarily European ancestry and 13,285 participants with primarily African ancestry from the Vanderbilt University Medical Center Biobank (BioVU). We examined the predictive properties of PGS_25OHD_, and two scores related to DBP concentration with respect to 1322 health-related phenotypes and 315 laboratory-measured phenotypes from electronic health records. In those with European ancestry: (a) the PGS_25OHD_ and PGS_DBP_ scores, and individual SNPs rs4588 and rs7041 were associated with both 25OHD concentration and 1,25 dihydroxyvitamin D concentrations; (b) higher PGS_25OHD_ was associated with decreased concentrations of triglycerides and cholesterol, and reduced risks of vitamin D deficiency, disorders of lipid metabolism, and diabetes. In general, the findings for the African ancestry group were consistent with findings from the European ancestry analyses. Our study confirms the utility of PGS and two key variants within the *GC* gene (rs4588 and rs7041) to predict the risk of vitamin D deficiency in clinical settings and highlights the shared biology between vitamin D-related genetic pathways a range of health outcomes.

While there is no doubt that vitamin D deficiency is causally associated with adverse bone outcomes (e.g., rickets in children, osteoporosis in adults), the influence of vitamin D on other health outcomes remains poorly understood (Holick, 2007). Cross-sectional observational studies often report an association between vitamin D deficiency (as defined by serum 25 hydroxyvitamin D [25OHD] concentration less than 25 nmol/L) and an increased risk of many different health outcomes, such as cancer, autoimmune disease, cardiovascular disease, and psychiatric disorders (Holick & Chen, 2008; Manson, Cook et al., 2019). In most instances, these associations merely reflect the well-accepted finding that poor general health can lead to low 25OHD concentration because of reduced outdoor activity and reduced exposure to bright sunshine. In addition, prior risk factors such as obesity and smoking can confound the apparent association between vitamin D deficiency and adverse health outcomes.

Recently, large, randomized controlled trials of vitamin D supplementation have not supported a causal role for vitamin D in health outcomes related to cancer, cardiovascular disease and bone outcomes (Chou et al., 2020; de Boer et al., 2019; LeBoff et al., 2020; Lucas & Wolf, 2019; Manson, Bassuk, Buring et al., 2020; Manson, Bassuk, Cook et al., 2020; Manson, Cook et al., 2019; Manson, Mora et al., 2019; Neale et al., 2022). These findings have lowered expectations about the role of vitamin D deficiency as a causal risk factor for many adverse health outcomes. However, because randomized controlled trials rarely extend beyond a few years, they are less able to detect exposure-risk relationships that have a long latency (e.g., suboptimal vitamin D status over many decades may contribute to the gradual loss of bone mineral density, and result in later-life osteoporosis; Heaney, 2003). In these scenarios, Mendelian randomization (MR) studies may be informative, as it is assumed that common genetic variants that influence phenotypes such as 25OHD concentrations would operate in a stable fashion across the entire lifespan. To date, MR studies related 25OHD have found evidence to support causal pathways with (a) multiple sclerosis (Jiang et al., 2021; Manousaki et al., 2017; Mokry et al., 2015; Rhead et al., 2016), (b) ovarian cancer (Ong et al., 2016), and (c) dyslipidemia (Revez et al., 2020). On the other hand, MR analyses by Revez and colleagues (2020) showed evidence supporting a causal effect of range of other health outcomes on 25OHD levels, but 25OHD only had an apparent causal effect on such health outcomes in the presence of horizontal (or biologically) pleiotropic variants, which influence both 25OHD concentration and health outcomes through independent pathways.

The analysis of other key elements of the vitamin D pathway may help clarify these findings. Recently, Albiñana and colleagues (2023) published a genomewide association study (GWAS) of the concentration of the vitamin D binding protein (DBP), a circulating protein involved in the transport and storage of 25OHD. Based on the genetic correlates of DBP, MR studies confirmed a strong positive and unidirectional association between DBP concentration and 25OHD concentration. Furthermore, there was a robust association between the genetic variants associated with higher DBP (higher polygenic score of DBP, PGS_DBP_) and higher (measured) concentration of 25OHD in the UK Biobank (UKB) sample. This study also used a set of genetic instruments adjusted for the prominent *cis*-protein quantitative trait loci (*cis*-pQTLs) in the *GC* gene (which encodes the DBP protein). Based on this subset of genetic variants, additional associations were found with a range of clinical phenotypes in the UKB, including reduced risk of hypertension, reduced pulse rate, reduced risk of gastritis and duodenitis, and an increased risk of allergic rhinitis and agranulocytosis). To the best of our knowledge, no studies have used both the GWAS findings from 25OHD and DBP to help clarify the role of vitamin D status across a wide range of health outcomes.

Phenome-wide association studies (PheWAS) and laboratory-wide association studies (LabWAS) (Goldstein et al., 2020) have the ability to explore the associations between (a) the genetic correlates of potential risk factors such as 25OHD and DBP concentration, and (b) a wide range of disease and laboratory phenotypes in clinical settings (Dennis, Sealock, Straub et al., 2021; Denny et al., 2010; Wei et al., 2017). A previous PheWAS examined the association between a polygene risk score PGS for 25OHD based on 6 independent genetic loci and a wide range of phenotypes available in the UKB (Meng et al., 2019). This study found no evidence of an association between 25OHD concentration and over 900 different clinical outcomes, but the authors noted that the study may have lacked the power to detect small effect sizes. We had the opportunity to conduct a PheWAS using the more powerful GWAS based on the UKB (*n* = 417,580), which identified 143 independent variants (Revez et al., 2020). In addition, we used the GWAS findings related to DBP (*n*= 65,589, 26 independent variants) from Albiñana and colleagues (2023), which allowed us to look for convergent evidence from these two key vitamin D pathway components. The summary statistics from these two GWAS analyses were used to predict a wide range of diseases and laboratory phenotypes available within the Vanderbilt University Medical Center (VUMC) electronic health record (EHR) in conjunction VUMC’s DNA repository, BioVU. Importantly, the VUMC cohort also represents a healthcare-seeking population, compared to the volunteer ascertainment of UKB, which provides additional opportunities to investigate the relationship between Vitamin D and illness across the medical phenome.

## Methods

### Study Population and Data Access Approval

Data for this study were obtained with permission from the Vanderbilt University Medical Center Biobank (VUMC BioVU) DNA databank in conjunction with the de-identified version of the VUMC EHR called the Synthetic Derivative. The study was approved by the VUMC IRB (IRB#190418). The study population included only patients genotyped on the Illumina Expanded Multi-Ethnic Genotyping Array (MEGAex). The database includes demographics, vital measurements, ICD9 and ICD10 codes, Current Procedural Terminology (CPT) codes, laboratory test results, medications, and clinical notes recorded from 1994 to 2021. Detailed information about BioVU’s data management and quality control, ethical considerations, and continuing patient engagement has been previously published (Bowton et al., 2014; Denny et al., 2010; Pulley et al., 2008; Ritchie et al., 2010; Roden et al., 2008). Of note, date shifting within a 1-year timeframe was adopted as a strategy to reduce potential identifiability. While dates are shifted by a consistent number of days within an individual’s medical record (i.e., birthday and all visits are shifted by the same number of days), the selected interval for the date-shifting differs between individuals. This practice limits our ability to detect seasonal associations with 25OHD concentrations because we lack precise dates for laboratory testing and code assignment.

### Genotyping and Quality Control

Genotypes for 94,474 individuals who received care at VUMC were obtained through BioVU. Genotypes were measured on the MEGAex array (Zhao et al., 2018), and ancestral clusters for individuals of inferred European or African ancestry were selected as previously described (Dennis, Sealock, Straub et al., 2021). Genotyping data within each ancestry group were imputed and underwent quality control checks as previously described. Briefly, European and African ancestry boundaries were calculated using Eigenstrat (Price et al., 2006). Data were imputed using the Michigan Imputation Server with the Haplotype Reference Consortium reference panel (McCarthy et al., 2016). Genotyping data was then subjected to a series of ancestry-specific QC filters, including minor allele frequency <0.05, imputation quality R^2 <0.3 thresholding, and π <0.2. The resulting dataset contained 6,360,678 variants from 66,917 people of European ancestry and 12,897,448 variants from 13,329 people of African ancestry.

We filtered samples to only those individuals with complete data on EHR reported sex and median age in the database (respectively 66,482 and 13,285 for European and African ancestry individuals). From these subsets we calculated the principal components (PCs) of genetic ancestry on a randomly selected subset of 250,000 SNPs using Flash PCA (Abraham & Inouye, 2014) and an in-house script (Abraham et al., 2017).

### Phenotype Data

#### PheWAS.

Phenotypic data were represented using phecodes generated by hierarchical clustering of related ICD codes (Denny et al., 2013). ICD-9 and 10 codes were mapped to 1664 phecode categories according to the Phecode Map v1.2 (https://phewascatalog.org/phecodes), as implemented in the PheWAS R package v0.12 (Carroll et al., 2014). Patients were assigned to the case group for a given phecode if they had at least two different ICD-9 or 10 codes that mapped to a given phecode, or if they had at least two separate occurrences (i.e., on different days) of a single ICD-9 or 10 code that mapped to the given phecode, both of which are validated strategies to improve the positive predictive value of phecodes (Denny et al., 2013). The control group excluded patients with only one component ICD-9 or 10 code, or with one or more ICD-9 or 10 codes that mapped to related phecodes (as defined by the Phecode Map v1.2).

#### LabWAS.

We used the previously described QualityLab and LabWAS pipelines to perform quality control and analysis of quantitative clinical laboratory (lab) tests data in the EHR (Dennis, Sealock, Straub et al., 2021). We extracted data on all lab tests collected in the routine clinical care of VUMC patients, resulting in data from 939 lab tests after the QualityLab pipeline was applied (Dennis, Sealock, Straub et al., 2021). SNP-based heritability of lab values was previously calculated and described in detail. As we are using polygenic risk scores to predict lab values, we restricted the analysis to tests with a non-zero estimated SNP-based heritability. This resulted in 318 labs available for analysis. In this primary analysis, we used the median lab values adjusted for cubic splines of median age at lab ascertainment (4 knots). We transformed lab values to fit the normal distribution to improve the performance of the linear regression models (McCaw et al., 2020). We applied the rank-based inverse normal quantile transformation (RINT) to all labs, which ensured trait normality by replacing the value of each observation with its quantile from the standard normal distribution.

Vitamin D can be measured clinically in a variety of forms. Overall vitamin D status is routinely assessed by assaying the transport and storage forms such as 25 hydroxyvitamin D3 and the closely related 25 hydroxyvitamin D2. Typically, the more abundant form, the D3 type, is the product of actinic pathways (i.e., the action of ultraviolet light on the skin). Both D3 and D2 can be obtained via supplements. The active hormonal form of vitamin D is 1,25 dihydroxyvitamin D (1,25OHD; either D2 or D3), which has a short half-life and is typically measured in picogram level concentrations. The assays for 25OHD and 1,25OHD were based on chemiluminescent magnetic microparticle immunoassays or quantitative chemiluminescent immunoassays respectively. The VUMC pathology laboratory participates in quality-assurance programs organized by DEQAS (the Vitamin D External Quality Assessment Scheme) and the National Institute of Standards and Technology (NIST; Dai et al., 2018). Here, we have included measurements for 25OHD by two different assays (25OHD_a2, *n* = 9,472; 25OHD_a3, *n* = 9,450) and 1,25OHD by three different assays (1,25OHD_a1, *n* = 18,247; 1,25OHD_a4, *n* = 3,227; 1,25OHD_a5, *n* = 2,672).

### Statistical Analysis

#### Polygenic score model training.

We generated several PGSs based on GWAS of 25OHD and DBP concentration. For 25OHD, we used the original 25OHD GWAS summary statistics reported by Revez et al. (2020). An additional GWAS of 25OHD was conducted in a sample of 8306 UKB participants with 25OHD concentrations available and genetically inferred predominant African ancestry. Ancestry was inferred based on a two-step approach described elsewhere (Wang et al., 2020). GWAS was conducted as described in Revez et al. (2020). Briefly, 25OHD concentrations were normalized with RINT and genetic variants were tested for association with RINT 25OHD using fastGWA (Jiang et al., 2019). Covariates included in the model were age, sex, month of assessment, supplement intake, and the first 10 within-ancestry PCs.

For DBP, we used the two scores provided by Albiñana et al. (2023), based on neonatal dried blood spots from the iPSYCH case-cohort sample (*n* = 65,589; Pedersen et al., 2018). The first score (PGS_DBP_), which is based on the entire genome, is dominated by the very large effect *cis*-pQTLs within the *GC* gene (which encodes the DBP protein). The second score (PGC_DBP_GC_) excludes variants within the *GC* gene and is better able to identify *trans*-pQTLs variants. The iPSYCH sample did not have sufficient sample size with African ancestry to generate ancestry-specific DBP-summary statistics.

All PGSs were calculated with PRS-CS (Polygenic Risk Score – Continuous Shrinkage), a Bayesian polygenic prediction method that imposes continuous shrinkage priors on SNP effect sizes (Ge et al., 2019). These priors can be represented as global-local scale mixtures of normals, which allow the model to flexibly adapt to differing genetic architectures and is computationally efficient. The shrinkage parameter was automatically learnt from the data (i.e., using PRS-CS-auto). SNP effect estimates were obtained from GWAS summary statistics, and the score was calculated using a linkage disequilibrium reference panel from 503 European samples from the 1000 Genomes Project phase 3 (1000 Genomes Project Consortium et al., 2015) for the European and African ancestry analyses. For the score generated using the GWAS summary statistics for 25OHD from samples of predominantly African ancestry, the shrinkage parameter was set to 1e-2 due to the small GWAS sample size and the score was calculated using a linkage disequilibrium reference panel from 661 African ancestry samples from the 1000 Genomes Project phase 3 (1000 Genomes Project Consortium et al., 2015). PGS estimates were scaled to have a mean of zero and a standard deviation (*SD*) of 1 within ancestry strata before testing for association with any outcome variables.

#### LabWAS of PGS_25OHD_, PGS_DBP_ and PGS_DBP_GC_.

After QC, we applied RINT to the median (across longitudinal measures within a person) lab values, to account for skewness and non-normality in the subsequent LabWAS. In this analysis, we tested the association between the predictor variables (PGS_25OHD,_ PGS_DBP_ and PGS_DBP_GC_) against all heritable clinically measured laboratory tests. Additionally, we imposed a minimum sample size requirement of 100 for a laboratory test to be included in the LabWAS analysis, bringing the number of labs tested in each scan to 315 in the European ancestry set and 230 in the African ancestry set. We examined the influence of each of the three PGS on each of the validated LabWAS variables controlling for sex, median age across all ICD codes in medical record, and the top 10 principal components to adjust for genetic ancestry. Results are reported as beta coefficients and their standard errors per *SD* increase in the PGS. The Bonferroni-corrected threshold for statistical significance across labs for the European ancestry samples was 0.05/315 = 1.59e-04 and for the African ancestry samples was 0.05/230 = 2.17e-4 (based on the number of labs tested).

#### PheWAS of PGS_25OHD_, PGS_DBP_ and PGS_DBP_GC_.

The PheWAS analysis was conducted using the PheWAS R package v0.12 (Carroll et al., 2014). As with LabWAS, we required phecodes to include at least 100 cases (leading to 1322 tested phecodes in the European ancestry set, 688 in the African ancestry set), and we included covariates for sex, median age, and the first 10 PCs of estimated from genetic data. Results are reported as odds ratios (*OR*s) and their 95% confidence intervals (CIs) *SD* (either 25OHD or DBP concentrations) increase in each of the three PGS scores. The Bonferroni-corrected threshold for statistical significance across all tested phecodes was 0.05/1,322 = 3.78× 10^−5^ for the European ancestry set and 0.05/688 = 7e-5 for the African ancestry samples.

#### Post-hoc analyses of PGS_DBP_ and PGS_DBP_GC_ PheWAS findings.

The study by Albiñana et al. (2023) included PheWAS analyses of *PGS*_*DBP*_ *and PGS*_*DBP_GC*_ based on the UKB, examining 25OHD concentration and a subset of UKB phenotypes (i.e., 1149 phenotypes, including 1027 diseases and a range of anthropometric, brain imaging and infectious disease antigens phenotypes). Based on the findings from the current study, we attempted to replicate selected findings in the other UKB phenotypes not examined in the earlier study. The PheWAS analysis was conducted in the UKB using the same models as outlines in Albiñana et al. (2023). The quantitative traits were normalized using RINT with mean zero and variance 1. The PRSs were generated using SBayesR (Lloyd-Jones et al., 2019) with the reference LD matrix estimated from 1,145,953 HapMap3 SNPs in the UKB. PRSs were computed for 348,501 individuals of European ancestry. The individuals were genetically unrelated (relationship < .05). The covariates included in the model were sex, age and the first 20 PCs.

#### The influence of rs4588 and rs7041 on PheWAS and LabWAS.

In addition to the polygene scores, we examined the influence of two missense variants with the *GC* gene (rs4588, rs7041) on the variables of interest. Albiñana et al. (2023) had previously demonstrated that the rs7041 variant explained 54% of the variance of DBP concentration in neonatal dried blood spots. For the individual SNPs, we examined an additive model (i.e., 0, 1, 2 coding for effect allele).

## Results

Our analyses included 88,019 BioVU patients of European (*n* = 66,483) or African ancestry (*n* = 13,285). In the European ancestry (EA) sample, 56% of patients were female and the mean age was 48.71 years. In the African ancestry (AA) sample, 61% of patients were female and the mean age was 38.6 years. See Table 1 for additional characteristics of patients included.

### European Ancestry — PGS_25OHD_

**Figure 1.**
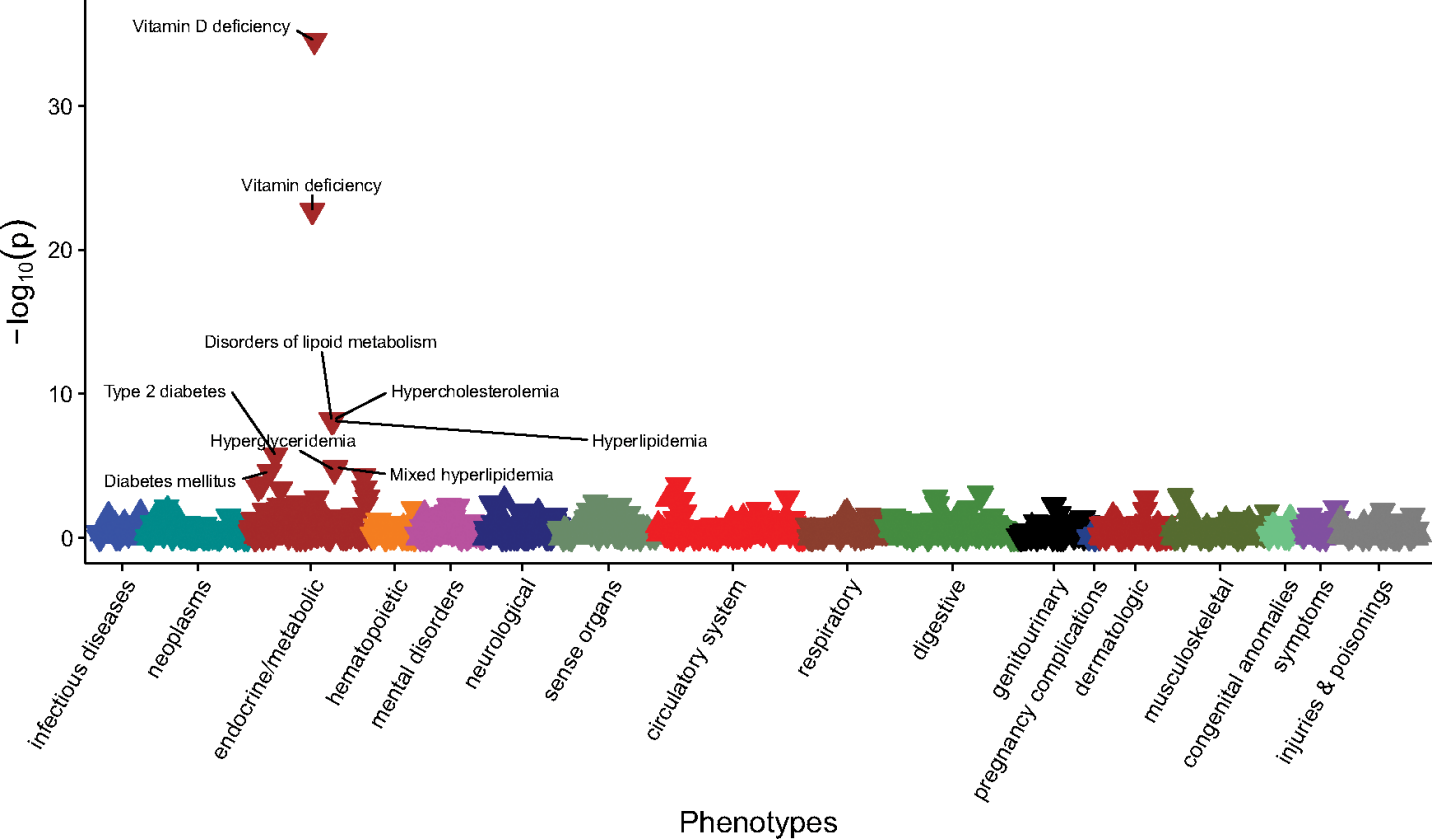
The association between PGS_25OHD_ and disease phenotypes in individuals with primarily European ancestry (*n* = 66,482). Note: Associations for 1322 phenotypes are shown. On the x-axis, the phenotypes clustered according to broad phenotype categories represented by different colors. *P* values are shown on the y-axis, with upturned triangles representing positive associations and downturned triangles representing negative associations. The top phenotypes with *p* values exceeding the Bonferroni multiple testing threshold (*p* < 3.78e-5), are labeled. Full details are provided in Supplementary data 1.

With respect to PheWAS (i.e., clinical phenotypes) in those with European ancestry, higher PGS_25OHD_ was associated (as expected) with lower odds of vitamin D deficiency (*OR* = 0.84, 95% CI [0.82, 0.86]; *n* cases = 5768, *n* controls = 45,960). Within the phenotypes that met the Bonferroni-adjusted threshold, of the nine top phenotypes (Figure 1), five were associated with altered lipid concentrations (e.g., reduced odds of hypercholesterolemia, *OR* = 0.92, 95% CI [0.90, 0.95]; *n* cases = 6925, *n* controls = 41,747). Two of the top nine phenotypes were related to a reduced risk of diabetes (e.g., reduced odds of Type 2 diabetes, *OR* = 0.95, 95% CI [0.93, 0.97], *n* cases = 10,202, n controls = 46,320) (Supplementary data 1).

**Figure 2.**
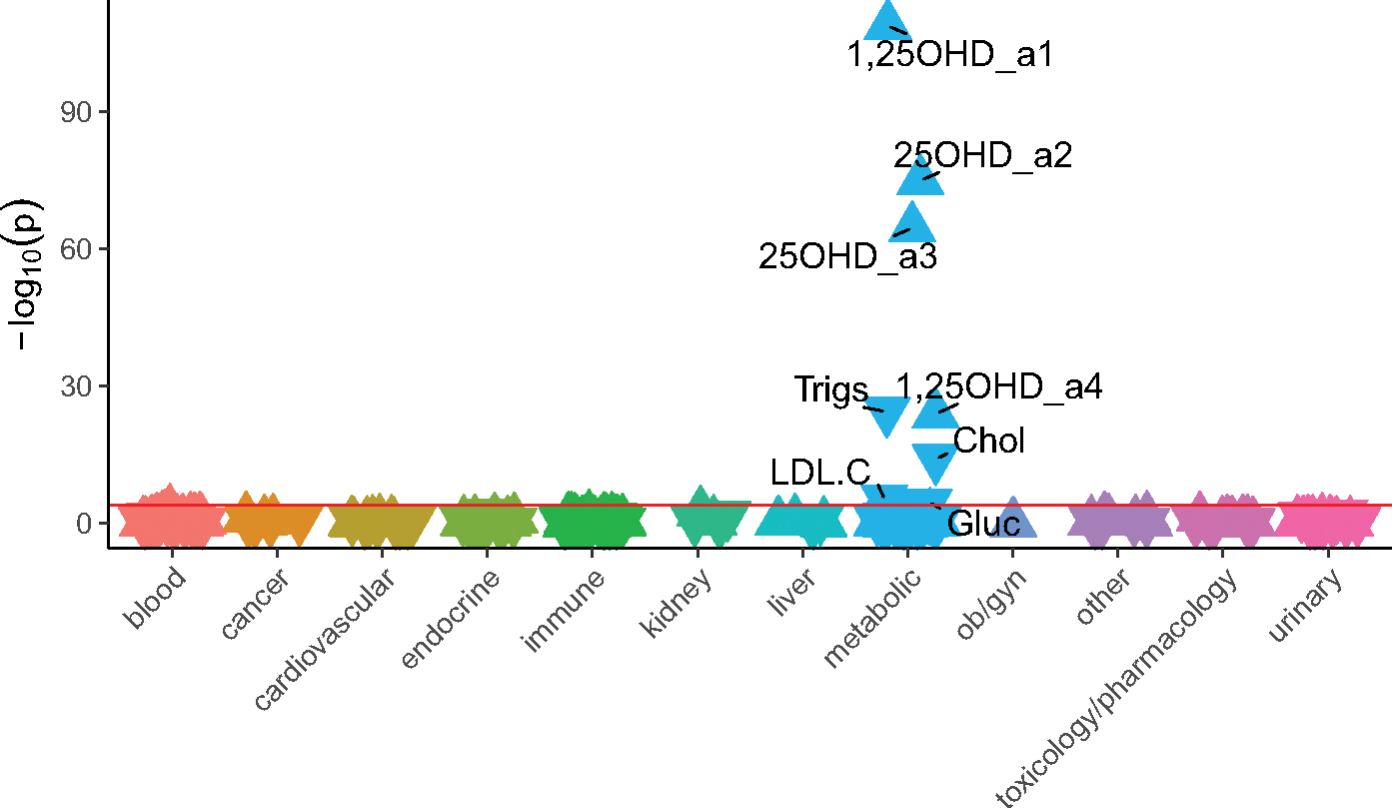
The association between PGS_25OHD_ and laboratory results, in individuals with primarily European ancestry (*n* = 66,482). Note: Associations for 315 laboratory results are shown. On the x-axis, the laboratory tests are clustered according to broad organ or pathology categories, represented by different colors. *P* values are shown on the y-axis, with upturned triangles representing positive associations and downturned triangles representing negative associations. Laboratory tests with p-values exceeding the Bonferroni multiple testing threshold (*p* < 1.59e-04), shown as a pink horizontal reference line, are labeled. Full details are provided in Supplementary data 2. 25OHD_a2 and 25OHD_a3 are two different types of 25 hydroxyvitamin D assays. 1,25OHD_a1 and 1,25OHD_a4 are two different types of 1,25 dihydroxyvitamin D assays. Trigs, triglycerides; Chol, cholesterol, LDL.C, low density lipoprotein cholesterol; Gluc, glucose.

LabWAS results (Figure 2) were consistent with the clinical diagnoses, with higher PGS_25OHD_ associated with both increased 1,25OHD concentration (β = 0.16, 95% CI [0.14, 0.17], *n* total = 18,247, *r*^2^ = .03) and increased 25OHD concentration (β = 0.18, 95% CI [0.16, 0.20]; *n* total = 9472, *r*^2^ = .03). Laboratory tests related to the measurement of cholesterol (β = −0.04, 95% CI [−0.05, −0.03], *n* total = 30,329, *r*^2^ = .002) and triglycerides (β = −0.06, 95% CI [−0.07, −0.05], *n* total = 30, 534, *r*^2^ = .003) had small but significant inverse associations with PGS_25OHD_, in keeping with the disease phenotypes described above. Finally, higher PGS_25OHD_ was associated with a small but significant reduction in glucose concentration (β = −0.015, 95% CI [−0.02, −0.008], *n* total = 62,280, *r*^2^ = .0003) (Supplementary data 2).

### European Ancestry — PGS_DBP_ and PGS_DBP_GC_

**Figure 3.**
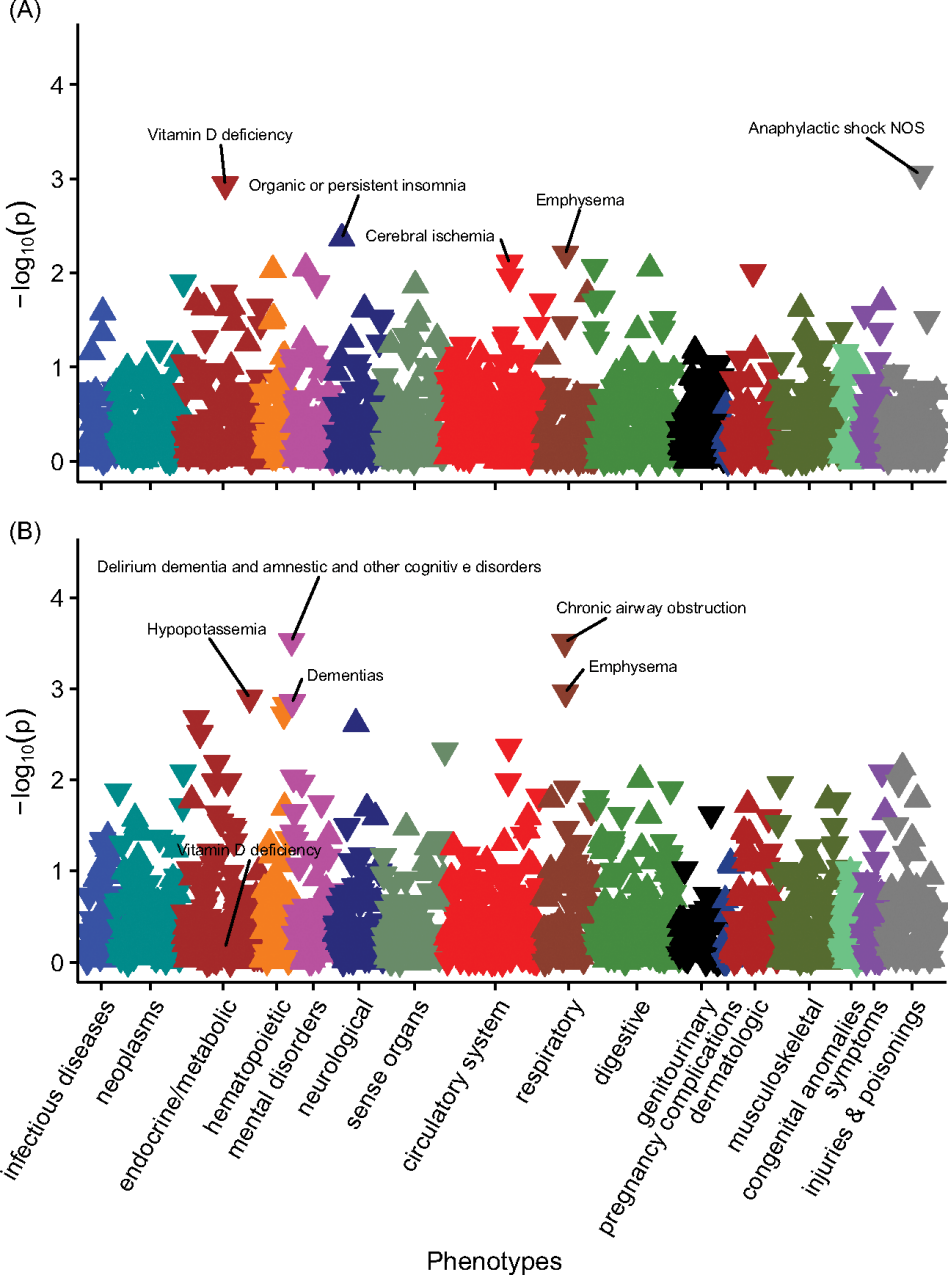
The associations between PGS_DBP,_ PGS_DBP_GC_ and disease phenotypes in individuals with primarily European ancestry (*n* = 66,482). Note: Panel A, PheWAS for PGS_DBP._ Panel B, PGS_DBP_GC._ Associations for 1322 phenotypes are shown. On the x-axis, the phenotypes clustered according to broad phenotypes represented by different colors. *P* values are shown on the y-axis, with upturned triangles representing positive associations and downturned triangles representing negative associations. The five phenotypes with the smallest *p* values are labeled; however, none of phenotypes exceeded the Bonferroni multiple testing threshold (*p* < 3.78e-05). In Panel B, Vitamin D deficiency is also labeled for reference. Full details are provided in Supplementary Data 3 and 5.

**Figure 4.**
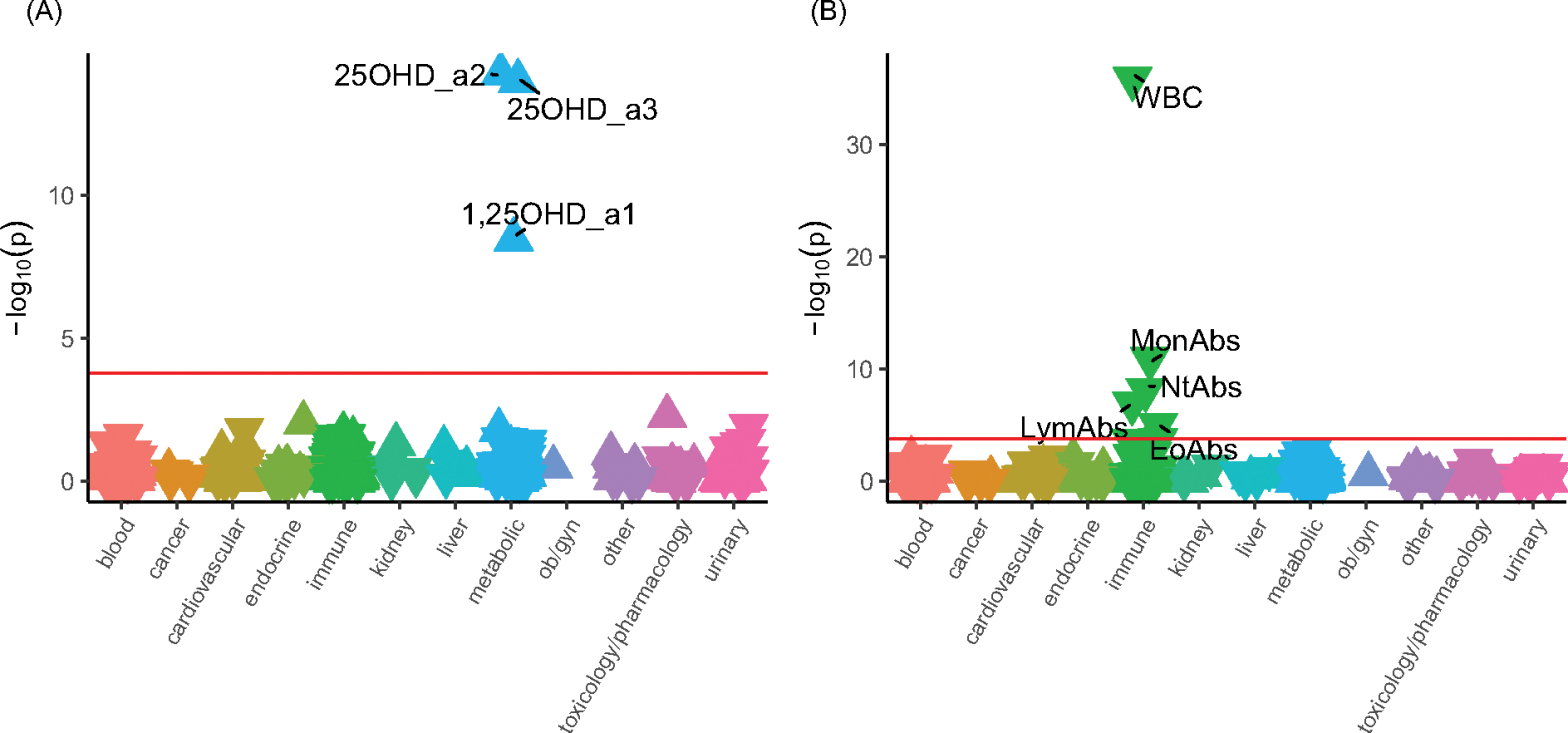
The associations between PGS_DBP,_ PGS_DBP_GC_ and laboratory measures in individuals with primarily European ancestry (*n* = 66,482). Note: Panel A, LabWAS for PGS_DBP._ Panel B, LabWAS for PGS_DBP_GC_. Associations for 315 laboratory results are shown. On the x-axis, the laboratory tests are clustered according to broad organ or pathology categories, represented by different colors. *P* values are shown on the y-axis, with upturned triangles representing positive associations and downturned triangles representing negative associations. Laboratory tests with *p* values exceeding the Bonferroni multiple testing threshold (*p* < 1.59e-04), shown as a pink horizontal reference line, are labeled. 25OHD_a2 and 25OHD_a3 are two different types of 25 hydroxyvitamin D assays. 1,25OHD_a1 and 1,25OHD_a4 are two different types of 1,25 dihydroxyvitamin D assays (see Methods). WBC, leukocytes (#/volume) in blood by automated count. LymAbs, lymphocytes (#/volume) in blood by automated count. MonAbs, absolute count of monocytes. NtAb, absolute count of neutrophils. EoAb, absolute count of eosinophils. Full details are provided in Supplementary data 4 and 6.

No PheWAS associations with PGS_DBP_ exceeded the Bonferroni adjusted *p*-value threshold in those with European ancestry (Figure 3). However, vitamin D deficiency was nominally significant (*OR* = 0.96, 95% CI [0.93, 0.98], *n* cases = 5768, *n* controls = 45,960) (Figure 4, Supplementary data 3).

With respect to LabWAS, in those with European ancestry, the two PGS related to DBP identified distinct findings (Figure 4). For PGS_DBP_ (which is strongly influenced by *cis*-pQTLs within the *GC* gene, which encodes for the DBP protein), there were small but significant associations with both 25OHD (e.g., β = 0.08, 95% CI [0.06, 0.10], *n* total = 9472, *r*^2^ =.006), and 1,25OHD (β = 0.04, 95% CI [0.03, 0.06], *r*^2^ = .002) (Supplementary data 4).

For the PGS_DBP_GC_ (which adjusts for variants within the *GC* gene to identify *trans*-pQTLs), there were no significant findings in the PheWAS analyses (Supplementary data 5). However, for the PGS_DBP_GC_ LabWAS analyses, we found small but significant reductions in white blood cell counts (leukocytes/lymphocytes, monocytes, neutrophils, eosinophils). For example, leukocyte counts were reduced in those with higher PGS_DBP_GC_ values (β=−0.044, 95% CI [−0.051, −0.037], *n* total = 64775, *r*^2^ = .002) (Supplementary data 6). As post-hoc analyses, we examined blood count related phenotypes in the UKB and confirmed a reduction in a range of comparable blood count related variables (Supplementary data 7). For example, higher PGS_DBP_GC_ values were significantly associated with reduced lymphocyte (i.e., leukocyte) count with a similar effect size as found in the main analysis (β = −0.039, 95% CI [−0.042, −0.035], *n* total = 291,968, *r*^2^ = .002).

### African Ancestry — PGS_25OHD_

We performed the PheWAS and LabWAS of the primarily African ancestry sample using summary statistics derived from the UKB African-ancestry population. The African ancestry derived PGS_25OHD_ identified one significant PheWAS finding, with higher genetically predicted 25OHD concentration being associated with a reduced risk of type 2 diabetes with renal manifestations (*OR* = 0.61, 95% CI [0.49, 0.78], *n* cases = 589, *n* controls = 9455). With respect to LabWAS findings, none were significant based on the Bonferroni-adjusted threshold (Supplementary data 8 and 9).

We also conducted the PheWAS and LabWAS of primarily African ancestry individuals using the PGS_25OHD_ trained on European derived summary statistics. Despite the much larger discovery sample size, no association exceeded the Bonferroni-corrected *p*-value threshold, but several of the diagnoses that associated with PGS_25OHD_ in the larger European target sample were nominally significant (*p* < .05) in the African ancestry target sample. For example, within the top 16 hits for the PGS_25OHD_ LabWAS analyses, three were for vitamin D-related measures (i.e., 25OHD or 1,25OHD). Those with higher PGS_25OHD_ scores had higher concentration of 1,25OHD (β = 0.05, 95% CI [0.02, 0.09], *n* total = 3279, *r*^2^ = .003). Also in the top 16 were two measures related to cholesterol (e.g., cholesterol [mass/volume] in serum or plasma, β = −0.04, 95% CI [−0.07, −0.02], *n* total = 5979, *r*^2^ = 0.002) (Supplementary data 10 and 11).

### African Ancestry — PGS_DBP_ and PGS_DBP_GC_

With respect to PGS_DBP_ and PGS_DBP_GC,_ we were restricted to using the PGS based on the original European-ancestry derived summary statistics. Based on these PGS scores, there were no significant PheWAS findings; however, the top hit for PGS_DBP_GC_ was a nominally significant protective finding for multiple sclerosis (*OR* = 0.76, 95% CI [0.65, 0.90], *n* cases = 159, *n* controls = 10,501). With respect to PGS_DBP_ and PGS_DBP_GC_ LabWAS findings, there were no significant findings; however, there was a small, nominally significant association between PGS_DBP_ and 25OHD concentration (β = 0.09, 95% CI [0.002, 0.169], *n* total = 473, *r*^2^ = .008). Full details of these analyses can be found in Supplementary data 12, 13, 14 and 15.

### The Influence of rs4588 and rs7041 on PheWAS and LabWAS Variables

The allele frequencies for rs4588 and rs7041 in the BioVU sample are shown in Supplementary Table 16. The presence of the G allele in rs4588, and the C allele in rs7041, were associated with higher concentration of 1,25OHD in both the European and African ancestry groups (Supplementary data 16).

With respect to PheWAS, in the European ancestry sample, for the two individual SNPs within the *GC* gene, rs4588 was significantly associated with the clinical diagnosis of Vitamin D deficiency (rs4588, *OR* = 0.86, 95% CI [0.83, 0.90], *p* = 1.98E-11, *n* cases = 5767, *n* controls = 45,944). rs7041 also had a significant association with Vitamin D deficiency (rs7041, *OR* = 0.90, 95% CI [0.87, 0.94], *p* = 1.77E-7, *n* cases = 5763, *n* controls = 45,935). However, there were no significant findings in the African ancestry group (Supplementary data 17, 18, 19 and 20). With respect to LabWAS in the European ancestry group, both individual SNPs were significantly associated with both 25OHD concentration and 1,25OHD (e.g., rs4588 and 25OD_a3, *n* total = 9450, β = 0.22, *SE* = 0.15, *p* = 4.36E-46; rs4588 and 1,24OHD_a1, *n* total = 18,247. β = 0.15, *p* = 1.25E-44. Supplementary data 21, 22, 23 and 24). With respect to the African ancestry sample, rs4588 was nominally significantly associated with both 25OHD and 1,25OHD, while rs7041 was only nominally significantly associated with 1,25OHD.

## Discussion

It was reassuring that the most recently published PGS for 25OHD (Revez et al., 2020) was able to predict 25OHD concentration and vitamin D deficiency. This study confirms that the genetic loci associated with 25OHD *and* DBP concentrations also predict a wide range of medical conditions and laboratory measurements within electronic health records in a general hospital setting. For example, we found that this same PGS predicted the risk of several phenotypes previously linked to vitamin D in observational and MR studies, including dyslipidemia and diabetes. In addition, the genetic correlates of DBP concentration also predicted 25OHD and 1,25OHD concentrations, and were associated with a range of white blood cell related measures. We will expand on these findings below.

Of particular interest, our findings lend weight to the hypothesis that variants associated with 25OHD are horizontally (or biologically) pleiotropic (Hemani et al., 2018), and influence 25OHD concentration among other biological functions, such as lipid pathways. In analyses that excluded potentially horizontally pleotropic variants, Revez and colleagues (2020) identified a persistent association between genetically predicted higher 25OHD concentration and a lower risk of dyslipidaemia. Many of the variants identified by Revez and colleagues were in genes related to lipid and lipoprotein pathways (e.g., *DHCR7, APOE, APOC1, DOCK7, CELSR2, LIPC, PCSK9*). While the mechanisms linking lipid and vitamin D pathways are poorly understood, there is evidence that vitamin D can inhibit activity of *DHCR7*, which encodes a key enzyme that diverts 7-dehydrocholesterol away from vitamin D biosynthesis and converts it to cholesterol (Prabhu et al., 2016; Zou & Porter, 2015). Regardless of the precise biological mechanisms, there is now convergent evidence from MR (Revez et al., 2020) and the current PheWAS study linking low 25OHD concentrations to an increased risk of dyslipidemia and higher concentrations of (a) triglyceride, (b) cholesterol, and (c) low density lipoprotein cholesterol. However, randomized clinical trials of vitamin D supplements have not reported strong effects on these phenotypes within their study timeframes (Costenbader et al., 2019; Meng et al., 2020; Ohlund et al., 2020). Thus, the clinical implications of these findings should be treated cautiously.

Our study also found that variants associated with higher 25OHD were associated with a reduced risk of diabetes and plasma glucose concentration. There are several potential biological mechanisms that could underpin this association. Revez et al. (2020) found that PGS_25OHD_ predicted a range of behaviors measured in the UKB including indoor activities (negatively associated with ‘hours spent using a computer’) and outdoor activity (positively associated with ‘duration of walks’ and ‘duration of vigorous activity’). Thus, at least some of the predictive properties of PGS_25OHD_ may be mediated by genetic variants associated with behaviors that influence actinic production of vitamin D. These same variables may influence body mass index, and subsequent risk of type 2 diabetes. Thus, the association between PGS_25OHD_ and diabetes may operate via pathways other than a direct influence of 25OHD concentration on the risk of diabetes.

To the best of our knowledge, this work also provides the first evidence to show that the PGS for 25OHD predicts 1,25OHD concentration. Studies of 1,25OHD are challenging because the half-life of this small molecule is short compared to 25OHD (several hours compared to one to two weeks, respectively; Zerwekh, 2008), and the concentration of 1,25OHD is tightly controlled by parathyroid hormone and calcium homeostasis. Several factors can uncouple the association between 25OHD and 1,25OHD. It has been reported that in the presence of both vitamin D deficiency (i.e., low 25OHD concentrations), and low calcium concentration, 1,25OHD concentrations can rise sharply — thus, this molecule is not regarded as a reliable measure of overall vitamin D status (Holick, 2009). From a clinical perspective, data from randomized controlled trials found that the use of oral vitamin D supplements is associated with an increase in the concentration of 1,25OHD (Zittermann et al., 2015). Albiñana et al. (2023) tested bidirectional MR models between the genetic correlates of 25OHD and DBP concentrations. They found a unidirectional association, which supports the hypothesis that higher DBP concentration may extend the functional half-life of 25OHD. Of interest, the two individual SNPs within the *GC* gene (rs4588 and rs7041) were associated with both 25OHD and 1,25OHD in the LabWAS for the European-ancestry sample. Within the smaller African ancestry sample the two individual SNPs were nominally significantly associated with 1,25OHD (rs4588 also had a nominally significant association with 25OHD). These findings provide new insights into the genetic architecture of vitamin D metabolism.

The vitamin D binding protein has a range of biological functions in addition to the transport of 25OHD and 1,25OHD (e.g., T-cell response, C5a-mediated chemotaxis, macrophage activation; Bouillon et al., 2019). Albiñana et al. (2023) found evidence from MR that increased DBP concentration based on the GWAS findings adjusted for variants in the *GC* gene were associated with a reduced risk of rheumatoid arthritis and multiple sclerosis. While we found a nominal association between PGS_DBP_GC_ and multiple sclerosis in the African ancestry sample, these disorders were not confidently detected in the current study. We did, however, find a range of decreased white blood cell trait counts associated with PGS_DBP_GC_. Pleotropic variants may account for this finding. A missense variant in *SH2B3*, is both (a) a ‘master regulator’ influencing the concentration of over 50 plasma protein (Ferkingstad et al., 2021; Pietzner et al., 2021; Sun et al., 2018), and (b) associated with a range of hematological measurements and disorders (Morris et al., 2021). The active form of vitamin D (1,25OHD) is a potent driver of cellular differentiation (in keeping with other steroid hormones) and in the presence of vitamin D deficiency, the hematological cell lines may be less differentiated, which in turn may explain the decrease in mature cell counts (Medrano et al., 2018).

The genetic correlations of GWAS summary statistics can be difficult to interpret, as cases used to derive the summary statistic may have an increased risk of additional correlated phenotypes (compared to non-cases). For example, it is feasible that the individuals in the UKB who had lipid-related phenotypes also had low 25OHD as a consequence of their impaired health (e.g., diabetes, obesity), and the GWAS methodology and subsequent PheWAS studies may detect both the target and correlated phenotypes (previously referred to as the ‘phenotypic hitchhiking’ effect; Dennis. Sealock, Levinson et al., 2021). Regardless of these issues, the findings of our study lend weight to the hypothesis that vitamin D pathways and lipid-related phenotypes may have shared biological pathways.

Finally, despite a much-reduced discovery sample size, the PGS_25OHD_ based on African ancestry derived summary statistics, detected an association between PGS_25OHD_ and type 2 diabetes with renal manifestations in the primarily African ancestry target sample. Importantly, this compares to an absence of significant associations in the exact same target sample when using the PGS_25OHD_ trained on sumstats from a primarily European ancestry sample. These findings illustrate that the absence of associations in the latter analysis is largely due to underrepresentation in the European ancestry GWAS and strongly signal the need for more ancestrally diverse genetic research in general and in vitamin D genetic studies specifically (Sirugo et al., 2019).

The study has several strengths. The electronic health records used in this study included a large sample of patients, with extensive information on treated phenotypes and laboratory tests. The PGS instrument was based on a more powerful GWAS study compared to the previous (null) PheWAS (Meng et al., 2019). However, there were several important limitations. The discovery sample for 25OHD was based on the UKB, which is not representative of the general community (Fry et al., 2017). As a result, if selective process are associated with both the predictor and outcome variable, collider biases may be introduced (Munafo et al., 2018), which can subsequently lead to spurious associations. Our African ancestry sample was small, and we were not able to examine diverse ancestries beyond African and European ancestry groups. Ideally, variant imputation and PRS scores generation should be based on appropriate African ancestry samples. Thus, our results are unlikely to be generalizable to other ancestries. Additionally, the Vanderbilt health system is a tertiary referral center, and may not be representative of population-based samples. Lastly, private health insurance is required in most primary care clinics at VUMC, which further limits the socioeconomic diversity of the patient population.

## Conclusions

Genetic instruments designed to predict vitamin D status were shown to have face validity in the large sample of European and African ancestry patients treated in a specialist health setting. The polygene risk score for 25OHD predicted clinical vitamin D deficiency, and also predicted the concentration of the active form of vitamin D, 1,25 dihydroxyvitamin D. In addition, two missense SNPs within the *GC* gene (rs4588 and rs7041) independently predicted both 25OHD and 1,25OHD concentrations, and thus could act as informative genetic instruments in MR models. Other phenotypes associated with our predictors include lipid-related diagnoses and diabetes. These findings lend weight to the hypothesis that low vitamin D may contribute to these clinical features.

## Supplementary Material

Supplementary Tables

## Figures and Tables

**Table 1.** Counts and univariates statistics for key demographic variables of the European and African ancestry groups

	European ancestry	African ancestry
Sample size (*N*)	66,483	13,285
Female, *N* (%)	37,001 (55%)	8102 (61%)
Mean (*SD*) of the person-level median age in years across the EHR	48.71 (22.27)	38.60 (21.33)
Length of EHR in years, Median (Q1-Q3)	9.58 (3.64–15.49)	8.55 (3.43–14.87)
Number of ICD codes Median (Q1-Q3)	131 (47–315)	102 (36–261)
Density of ICD codes (# ICD codes/length of EHR) Median (Q1-Q3)	18.8 (8.16–48.12)	16.81 (7.23–42.41)

Note: EHR, electronic health record; *SD*, standard deviation; Q1-Q3, first and third quartile.

## List of abbreviations.

1,25OHD: 1,25 dihydroxyvitamin D
25OHD: 25 hydroxyvitamin D
DBP: vitamin D binding protein
EHR: Electronic health record
GWAS: genomewide association study
MR: Mendelian randomization
PGS: polygene scores
PheWAS: Phenome-wide association study
LabWAS: Laboratory-wide association study
RINT: rank-based inverse normal transformation
SNP: single nucleotide polymorphism
UKB: UK Biobank
